# Protein *S*-nitrosylation in plants under abiotic stress: an overview

**DOI:** 10.3389/fpls.2013.00373

**Published:** 2013-09-20

**Authors:** María C. Romero-Puertas, María Rodríguez-Serrano, Luisa M. Sandalio

**Affiliations:** Departamento de Bioquímica, Biología Celular y Molecular de Plantas, Estación Experimental del Zaidín, Consejo Superior de Investigaciones CientíficasGranada, Spain

**Keywords:** ****abiotic stress, nitric oxide, plant, post-translational modifications, *S*-nitrosylation

## Abstract

Abiotic stress is one of the main problems affecting agricultural losses, and understanding the mechanisms behind plant tolerance and stress response will help us to develop new means of strengthening fruitful agronomy. The mechanisms of plant stress response are complex. Data obtained by experimental procedures are sometimes contradictory, depending on the species, strength, and timing applied. In recent years nitric oxide has been identified as a key signaling molecule involved in most plant responses to abiotic stress, either indirectly through gene activation or interaction with reactive oxygen species and hormones; or else directly, as a result of modifying enzyme activities mainly by nitration and *S*-nitrosylation. While the functional relevance of the *S*-nitrosylation of certain proteins has been assessed in response to biotic stress, it has yet to be characterized under abiotic stress. Here, we review initial works about *S*-nitrosylation in response to abiotic stress to conclude with a brief overview, and discuss further perspectives to obtain a clear outlook of the relevance of *S*-nitrosylation in plant response to abiotic stress.

## INTRODUCTION

During their life, plants are continuously exposed to extremes in environmental conditions, in particular abiotic stress sources such as drought, high or low temperatures, high salinity, heavy metal exposure, or herbicides affecting plant development and production (Mittler, 2006; Sreenivasulu et al., 2007). Over 90% of the world’s arable lands are reportedly exposed to major environmental stresses (Pinho dos Reis et al., 2012). Plants use complex recognition and response mechanisms to protect themselves from environment-related changes (Yamaguchi-Shinozaki and Shinozaki, 2006). The effects of abiotic stress may be general or non-specific, such as growth inhibition, electrolyte leakage, and excess of reactive oxygen species (ROS), all of which could lead to cell death. Each type of stress, however, induces specific responses involved in plant acclimation to the particular stress (Kreps et al., 2002; Rizhsky et al., 2004). Therefore, determining the mechanism underlying plant stress tolerance and adaptation is a high priority to ensure plant fitness over a wider range of environmental conditions.

Nitric oxide (NO) is a ubiquitous inter- and intracellular signaling molecule found to be involved in a myriad of cellular functions in plants (Neill et al., 2008b; Mur et al., 2013). Having gained knowledge about its high reactivity and its ambivalent effect, depending on the rate/place of production, there is a need to explore how NO develops all these functions. NO regulates different processes by inducing gene transcription or activating secondary messengers (Besson-Bard et al., 2008; Palmieri et al., 2008; Gaupels et al., 2011). Moreover, NO controls diverse biological processes by directly altering proteins (Martinez-Ruiz et al., 2011). It is able to regulate enzyme activity through covalent post-translational modifications (PTMs) joining metal centers of the proteins and NO tends to affect cysteine and tyrosine residues of the proteins (*S*-nitrosylation and nitration, respectively) changing their activity, location, or aggregation state (Souza et al., 2008; Martinez-Ruiz et al., 2011). Protein* S*-nitrosylation, the incorporation of a nitroso group to a Cys thiol, has been established as a significant route through which NO transmits its global cellular influence, and as a broad-based mechanism for the post-translational regulation of most or all classes of proteins (Stamler et al., 2001; Lindermayr and Durner, 2009; Astier et al., 2011). In this review we will focus on the current state of knowledge regarding *S*-nitrosylation in plants grown under abiotic stress and the elucidation of the function of NO as a signaling mechanism in plant response to environmental modifications.

## NO AND ABIOTIC STRESS

Klepper (1979) demonstrated by the end of 1970s that herbicides treated soybean leaves release NO*_x_* (thought to be mainly NO). Then, in the 1990s, new works evidenced plant NO production in response to abiotic stress (Leshem and Haramaty, 1996; Leshem et al., 1998). Notwithstanding, the first confirmations presenting NO as a key signaling molecule were achieved in response to biotic stress (Delledonne et al., 1998; Durner et al., 1998). In parallel a number of reports on exogenous NO effects in plants were shown (Lamattina et al., 2003). From the very beginning the dual effects of NO as a promoter and inhibitor were seen to depend mainly on its concentration (Leshem and Haramaty, 1996; Beligni and Lamattina, 1999). Currently, many examples of the effect of NO donors on plant biology highlight the protective role of NO against abiotic stresses such as salinity, drought, heavy metals, or UV-radiation (Corpas et al., 2006; Tossi et al., 2012a). Special caution needs to be taken with results obtained with NO donors (Murgia et al., 2004), as each one has specific chemical characteristics that are determinant for the timing of NO release, in addition to features such as the pH of the media, temperature, or light (Ramamurthi and Lewis, 1997; Lamattina et al., 2003). It is therefore difficult to derive physiological conclusions with NO donor effects on plants, as it is hard to measure the real concentration of this molecule in the cell and identify specific targets for each one. Still, the pharmacological approach seems necessary until the mechanism of NO generation in plants can be better defined (Gupta et al., 2011; Mur et al., 2011). Some mutant plants with altered endogenous NO production have been described, involving different NO production pathways or inducing NO production indirectly (Mur et al., 2013). Thus, the use of a combined approach, genetic and pharmacological, would shed light on the functional relevance of NO in response to abiotic stress.

In the past decade, a number of articles have addressed plant endogenous NO production/reduction in a wide range of species, in response to different abiotic stresses (Gould et al., 2003; Corpas et al., 2006; Tossi et al., 2012a). Such studies entail technical problems in assessing the precise location and amount of NO *in planta *(Mur et al., 2013), and the timing and concentration of the stress. It is lacking: are key factors in NO production. Thus, one single stress type could produce contrary effects depending on the application time and strength of the stress. Meanwhile, there are non-technical problems due to the idiosyncrasy of plants, at least seven sources of NO have been described (Gupta et al., 2011), and more than one source could be involved in the response of a certain stress. Contradictory effects might also depend on the species, tissue analyzed, and developmental stage of the plant.

Heavy metals, and specifically Cd provide a clear example of this diversity of NO-timing and effects (see Bartha et al., 2005; Barroso et al., 2006; Rodriguez-Serrano et al., 2006, 2009; Besson-Bard et al., 2009; De Michele et al., 2009). It appears that initial peaks of NO in response to Cd could have to do with signaling functions involved in iron homeostasis and root growth (Besson-Bard et al., 2009).When Cd treatment is in excess (150 μM), NO may be related with programmed cell death (PCD) induction (De Michele et al., 2009), whereas in long-term Cd treatment (50 μM), NO is associated with an induced senescence process stemming from an excess of ROS and ethylene (McCarthy et al., 2001; Romero-Puertas et al., 2002; Rodriguez-Serrano et al., 2009).

Drought is one of the main stresses affecting crop production, and again the role of NO in plant response is not clear (see Leshem and Haramaty, 1996; Magalhaes et al., 2000; Zhao et al., 2001; Garcia-Mata and Lamattina, 2002; Gould et al., 2003; Zhang et al., 2007). An important point is that stomata closure/aperture is essential during drought stress, a process controlled by abscisic acid (ABA), and NO is needed during the ABA-induced stomatal closure of turgid leaves, but there is not such necessity under conditions of rapid dehydration. NO could therefore be involved in the fine tuning of stomata closure in turgid leaves that occurs in response to oscillations in the environment (Garcia-Mata and Lamattina, 2002; Neill et al., 2008a; Wilson et al., 2009).

The literature describes NO responses to other abiotic stresses such as extreme temperatures, salinity, mechanical damage, UV-B, ozone, or herbicides (Gould et al., 2003; Corpas et al., 2006; Neill et al., 2008a; Molassiotis et al., 2010; Tossi et al., 2012b; Sehrawat et al., 2013; Xie et al., 2013). Although much work remains to define the physiological function of this molecule in response to abiotic stress, progress is underway. The NO signaling mechanisms has been building up beginning with transcriptomic analysis (Besson-Bard et al., 2008). NO has also been shown to mediate in different hormone-regulated processes in plants such as salicylic acid (SA), ABA, auxins, ethylene, or DELLAs, and a cross-talk between NO and hormones has been described in response to environmental fluctuations that may involve second messengers such as Ca or kinases (Lamattina et al., 2003; Simontacchi et al., 2013). The existence of a feedback mechanism between NO and ROS has been demonstrated, and ROS/NO balance is an important factor for the fate of the cells, especially in response to abiotic stress in the context of antioxidant systems and ROS production (Neill et al., 2003, 2008b; Rodriguez-Serrano et al., 2009). A further line of study focuses on direct NO-dependent protein regulation, mainly through *S*-nitrosylation and nitration (Astier et al., 2011; Vandelle and Delledonne, 2011).

## *S*-NITROSYLATION UNDER ABIOTIC STRESS

A key feature of NO biology is the PTM of cysteine thiol to form nitrosothiols (*S*-nitrosylation; Stamler et al., 2001). The development of biotin-switch (BST) technology that overcomes the sensitivity of the nitrosothiol group ensures more rapid entry to the world of NO biology. This elegant approach, formulated in the Snyder laboratory, facilitates the identification of *S*-nitrosylated proteins *in situ* as well as *in vitro *(Jaffrey and Snyder, 2001). The BST is currently the most commonly used method, though other promising approaches that are based on the BST have been developed (Seth and Stamler, 2011). Both approaches to detect *S*-nitrosylation have been used in plants grown under abiotic stress, proteome-wide scale and analysis of specific proteins known to be involved in the response of the plant to the mentioned stress (**Table 1**; Astier et al., 2011). Basically, nitrosylating agents (mainly *S*-nitrosoglutathione; GSNO) were used in proteome-wide scale analysis to increase the number of *S*-nitrosylated proteins before facing the proteomic study (Lindermayr et al., 2005; Abat et al., 2008; Abat and Deswal, 2009; Palmieri et al., 2010; Ortega-Galisteo et al., 2012; Begara-Morales et al., 2013; Kato et al., 2013). Other studies have been made in plants grown under stress to pathogen challenge (Romero-Puertas et al., 2008; Maldonado-Alconada et al., 2011) and later on under abiotic stress (Abat and Deswal, 2009; Tanou et al., 2009; Fares et al., 2011; Lin et al., 2012; Camejo et al., 2013). Salinity is the best characterized abiotic stress with regard to *S*-nitrosylation. Forty-nine proteins differentially *S*-nitrosylated were found in *Citrus aurantium* leaves under salt stress. Interestingly, a link between *S*-nitrosylation and oxidative damages (carbonylation) was detected, somehow involved in the prevention of protein loss of function by carbonylation, especially under stress conditions (Tanou et al., 2009). Brief salt stress in *Arabidopsis* cell suspensions showed that NaCl modified the *S*-nitrosylation level of a small proportion of endogenously *S*-nitrosylated proteins (around 10%), suggesting that salt stress induced minor modulations of the *S*-nitrosylation pattern rather than major changes (Wawer et al., 2010; Fares et al., 2011). Interestingly, this lab adapted the method for detection of endogenous *S*-nitrosylated Cys (Fares et al., 2011). Deeper analysis in isolated organelles could contribute to finding new targets as yet undetected in total extracts; along this lines, a proteomic study was done in mitochondria from pea plants subjected to salt stress (Camejo et al., 2013). A reduction of the *S*-nitrosylation pattern was reported in both short and long-term salt treatment being greater in the latter (Camejo et al., 2013). During salt treatment, proteins from respiratory and photorespiratory pathways and, significantly, antioxidant enzymes changed their *S*-nitrosylation pattern (Fares et al., 2011; Camejo et al., 2013). Changes in the *S*-nitrosoproteome were studied under low temperature as well, with nine spots induced and eight spots reduced differentially identified as plant defense-related, photosynthetic, glycolytic, and signaling-associated mechanisms (Abat and Deswal, 2009). Another noteworthy study looked at the *S*-nitrosoproteome under high-light conditions from wild type (WT) and *noe1* (NO excess1) mutant rice, and showed 48 proteins differentially *S*-nitrosylated in the mutant, 10% related to environmental adaptation and 14% to redox homeostasis (Lin et al., 2012). Indeed, *noe1 *mutants have increased H_2_O_2_ and NO levels and display NO-dependent PCD under high-light conditions. The authors found glyceraldehyde 3-phosphate dehydrogenase (GAPDH) and thioredoxin (Trx) *S*-nitrosylated in *noe1* mutants but not in WT, suggesting a relation of these proteins with the control of light-mediated leaf cell death in rice (Lin et al., 2012), reportedly involved in cell death in animals.

**Table 1:** Plant proteins regulated through *S*-nitrosylation in response to abiotic stress.

Plant system	Abiotic stress	NO/GSNOR/SNOs	Number of proteins differentially *S*-nitrosylated	Activity affected by *S*-nitrosylation	Reference
*Arabidopsis thaliana *leaves	Hypoxia	Increase/–/–	1	AHb1	Perazzolli et al. (2004)
*Brassica juncea *seedlings	Low temperature (6 h)	–/–/increase	17: 9 up/8 down	Rubisco	Abat and Deswal (2009)
*Citrus aurantium *leaves	NaCl 150 mM (16 d)	–/–/decrease	49	–	Tanou et al. (2009)
*Nicotiana tabacum * (BY-2 cells)	NaCl 250 mM (0–60 min)	–/–/–	1	GAPDH^*^	Wawer et al. (2010)
*Arabidopsis thaliana *suspension cells	NaCl 100 mM (5 min)	–/–/–	5: 3 up/2 down	–	Fares et al. (2011)
*Antiaris toxicaria *seeds	Desiccation (6 d)	Increase/–/–	3	APX, GR, DHAR	Bai et al. (2011)
*Oryza sativa *seedlings (WT vs. *noe 1*)	High light (2 d)	–/–/increase	69^**^	GAPDH, Trx^***^	Lin et al. (2012)
*Pisum sativum *leaves	Cd 50 μM (2 weeks) 2,4-D 23 mM (72 h)	Decrease/decrease /= Decrease/increase/increase	2	CAT, GOX	Ortega-Galisteo et al. (2012)
*Pisum sativum *mitochondria	NaCl 150 mM (5 d) NaCl 150 mM (14 d)	=/increase/decrease Increase/increase/decrease	9newline 14	PrxII F	Camejo et al. (2013)

* The enzyme activity was not impaired *in vivo* following the exposure of BY2 cells to salt or DEA/NO but *in vitro*.

** Differentially *S*-nitrosylated in *noe1 *vs. WT, all of them under high-light conditions.

*** In this paper it is suggested that *S*-nitrosylation of GAPDH and TRX in *noe1* plants parallels the development of cell death in animal systems.

*S*-nitrosylation pattern of particular proteins involved in a specific stress have been studied in parallel. The first protein identified as undergoing *S*-nitrosylation was hemoglobin AHb1 from *Arabidopsis thaliana*, which reduces NO emission under hypoxic stress through the production of *S*-nitrosohemoglobin (Perazzolli et al., 2004). Then, GAPDH showed a transient increase in its *S*-nitrosylation level in a tobacco cell culture in response to salt stress. However, further analysis is needed to explore the physiological relevance of this change over the treatment period, as its interaction with the osmotic stress-activated protein kinase (NtOSAK) was not affected (Wawer et al., 2010). Antioxidant enzymes from the Asc-Glu cycle, ascorbate peroxidase, glutathione reductase, and dehydroascorbate reductase (APX, GR, and DHAR, respectively) have reduced their *S*-nitrosylated pattern in response to seed desiccation, thereby suggesting a regulation of antioxidant enzyme activities to stabilize H_2_O_2_ accumulation at an appropriate concentration and increasing seed tolerance to dehydration (Bai et al., 2011). *S*-nitrosylation level of the peroxisomal protein glycolate oxidase decreased under cadmium and 2,4-dichlorophenoxyacetic acid (2,4-D) treatments while no differences were found under 2,4-D in *S*-nitrosylation level of CAT. Also, a reduction of *S*-nitrosylated CAT under Cd treatment was observed but similar to the changes occurred in the total amount of this protein under Cd stress. These results point to a regulation of H_2_O_2_ level under these stress conditions by NO through the control of ROS sources and antioxidant defenses (Ortega-Galisteo et al., 2012). Additionally, phytochelatins (PCs) with a specific nitrosylation signature were found in *Arabidopsis* cells treated with Cd, suggesting an interference with the capacity of PC to chelate the metal (De Michele et al., 2009).

## CONCLUSIONS AND PERSPECTIVES

**FIGURE 1:**
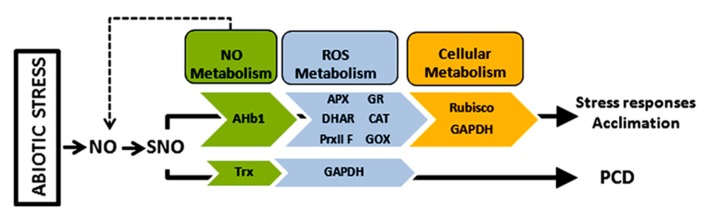
***S*-nitrosylated proteins under abiotic stress.** Proteins that change their *S-*nitrosylation pattern and activity in response to abiotic stress are related to NO, ROS, and cellular metabolism. AHb1, haemoglobin 1; APX, ascorbate peroxidase; CAT, catalase; DHAR, dehydroascobate reductase; GAPDH, glyceraldehyde 3-phosphate dehydrogenase; GOX, glycolate oxidase; Trx, thioredoxin. It is lacking: PrxII F, peroxiredoxin II F.

The identification of a number of plant proteins that change their *S*-nitrosylation pattern under abiotic stress is the starting point for the functional and biochemical characterization of *S*-nitrosylation in plants under such pathophysiological conditions (**Figure 1**). While the physiological relevance of *S*-nitrosylation have been shown with various proteins during plant–pathogen interactions (Spoel and Loake, 2011; Yu et al., 2012) this process has been poorly investigated under abiotic stress. Redox and oxygen metabolism-related proteins comprise an interesting group of targets of *S*-nitrosylation under abiotic stress, as these enzymes and the redox state of the cell play a key role in plant responses to environmental changes. A future challenge will be to unravel the NO-dependent control of these proteins, as a fine-tune regulation may exists in the NO/ROS balance to define the fate of the cell, especially under abiotic stress. Likewise important is the determination of NO-dependent interaction with hormones, especially those linked to plant responses to abiotic stress such as ABA (Roychoudhury et al., 2013), given that a link has been found between *S*-nitrosylation and auxins (Terrile et al., 2012). The regulation by *S*-nitrosylation of other protein PTMs that play a crucial role in cellular signaling, including phosphorylation, acetylation, or ubiquitylation, is an intringuing topic (Hess and Stamler, 2012). In this sense, initial data support the idea that *S*-nitrosylation could interfere with protein carbonylation under abiotic stress (Tanou et al., 2012).

Two important open questions regarding NO production can be summed up as “how and where”; especially in response to abiotic stress where the adverse condition is not as localized as in response to pathogens. The regulation of NO levels, and particularly its degradation, would be another topic of debate calling for study. Hemoglobin AHb1 has been shown to detoxify NO during hypoxic stress (Perazzolli et al., 2004), but its involvement in different abiotic stresses is still unknown. GSNO reductase (GSNOR) can control levels of GSNO, indirectly regulating *S*-nitrosylation-dependent signaling, though its participation in abiotic stress must be further explored. Finally, elucidation of the cellular distribution and characterization of nitrosylases and de-nitrosylases that may or may not involve Trx system is a matter essential for our understanding of the full scope of *S*-nitrosylation in plants under physiological and pathophysiological conditions.

## Conflict of Interest Statement

The authors declare that the research was conducted in the absence of any commercial or financial relationships that could be construed as a potential conflict of interest.
